# Feature Selection and Dimension Reduction of Social Autism Data

**Published:** 2020

**Authors:** Peter Washington, Kelley Marie Paskov, Haik Kalantarian, Nathaniel Stockham, Catalin Voss, Aaron Kline, Ritik Patnaik, Brianna Chrisman, Maya Varma, Qandeel Tariq, Kaitlyn Dunlap, Jessey Schwartz, Nick Haber, Dennis P. Wall

**Affiliations:** 1 Department of Bioengineering, Stanford University, Palo Alto, CA, USA; 2 Department of Biomedical Data Science, Stanford University, Palo Alto, CA, USA; 3 Department of Neuroscience, Stanford University, Palo Alto, CA, USA; 4 Department of Computer Science, Massachusetts Institute of Technology, Cambridge, MA, USA; 5 Department of Computer Science, Stanford University, Stanford, CA, USA; 6 Graduate School of Education, Stanford University, Palo Alto, CA, USA; 7 Department of Pediatrics, Stanford University, Palo Alto, CA, USA

**Keywords:** Autism, Diagnostics, Deep Learning, Feature Selection, Dimension Reduction

## Abstract

Autism Spectrum Disorder (ASD) is a complex neuropsychiatric condition with a highly heterogeneous phenotype. Following the work of Duda et al., which uses a reduced feature set from the Social Responsiveness Scale, Second Edition (SRS) to distinguish ASD from ADHD, we performed item-level question selection on answers to the SRS to determine whether ASD can be distinguished from non-ASD using a similarly small subset of questions. To explore feature redundancies between the SRS questions, we performed filter, wrapper, and embedded feature selection analyses. To explore the linearity of the SRS-related ASD phenotype, we then compressed the 65-question SRS into low-dimension representations using PCA, t-SNE, and a denoising autoencoder. We measured the performance of a multi-layer perceptron (MLP) classifier with the top-ranking questions as input. Classification using only the top-rated question resulted in an AUC of over 92% for SRS-derived diagnoses and an AUC of over 83% for dataset-specific diagnoses. High redundancy of features have implications towards replacing the social behaviors that are targeted in behavioral diagnostics and interventions, where digital quantification of certain features may be obfuscated due to privacy concerns. We similarly evaluated the performance of an MLP classifier trained on the low-dimension representations of the SRS, finding that the denoising autoencoder achieved slightly higher performance than the PCA and t-SNE representations.

## 1. Introduction

Autism Spectrum Disorder (ASD) is a complex developmental disability with a highly heterogeneous phenotype. ASD affects at least 214 million children worldwide, including one million children in the U.S. ten years of age and younger.^1^ Common behavioral traits associated with ASD include a struggle to make eye contact, recognize facial expressions, and engage in social interactions.^2^ Other behaviors often associated with ASD include repetitive behaviors, poor motor skills, and difficulty with language.^3^ ASD consists of several distinct and co-occurring symptoms.

The Social Responsiveness Scale, Second Edition (SRS) is a 65-item questionnaire filled out by a caregiver or teacher and designed to provide a metric for assessing social deficits and ASD severity in individuals 4 to 18 years old.^4^ The scale measures social responsiveness on five sub-scales: social awareness, social cognition, social communication, social motivation, and restrictive interests and repetitive behaviors. High scores indicate increasing social deficit and ASD severity. The scale is also used to assess ASD in research settings, e.g., as an outcome measure in digital therapies for ASD treatment.^5,6^

Duda et al.^7,8^ have performed item-level question selection on the SRS to identify questions which may provide the most predictive power in determining ASD classification while eliminating uninformative questions. For classification with relatively small training samples and high dimensionality, as is the case with ASD questionnaires, feature selection is essential for avoiding overfitting and improving overall classifier performance. Duda et al. identified the following top-ranking features for predicting ASD diagnosis: trouble with the flow of normal conversation, difficulty with changes in routine, lack of appropriate play with peers, difficulty relating to peers, atypical and inconsistent eye contact, and being regarded as ‘odd’ by other children.^7^ Bone et al. performed feature selection when combining questions from the SRS as well as the Autism Diagnostic Interview-Revised (ADI-R)^9^ using greedy forward-feature selection, finding that questions from the ADI-R can sometimes be more useful for distinguishing ASD from controls.^10^ Nevertheless, Duda et al. have used the top-ranking questions from the SRS to crowdsource parental responses to instrument-derived versions of the top-15 SRS features, achieving an AUC of 0.89.^8^

Identifying salient behavioral features that overlap when predicting diagnostic outcomes is pertinent to preserving privacy in digital diagnostics and interventions. When collecting digital data from a sensitive population, such as pediatric groups or individuals with psychiatric conditions, the digital data streams may require obfuscation in order to satisfy clinical or legal privacy requirements as well as the desire of the patient or caregiver to consent to the data collection process. Such obfuscation of data, however, may degrade the performance of diagnostic classifiers. Identifying redundant features can ameliorate or minimize these concerns by providing alternative areas of focus for data collection and quantification.

In order to identify new behavioral targets for ASD diagnostics and demonstrate the potential to identify overlapping features for privacy-preservation of these features, we expand the dataset of SRS questionnaires to 16,527 individuals, building upon previous work using 3,417 cases and controls combined.^8^ Using deep learning, we validate the predictive power of subsets of questions from the SRS for determining the diagnostic classification of a large pediatric population. We perform filter, wrapper, and embedded feature selection analyses to identify top-ranking questions as well as redundancies between features. Many of the most salient features validate those identified previously by Duda et al. while new additional features also become prominent. In addition, to explore the linearity of the social ASD space, we reduce the dimensionality of the 65-item SRS questionnaire into low-dimension representations using PCA, t-SNE, and a denoising autoencoder.

Our primary goal for this work is to identify new behavioral targets for ASD diagnostics via the largest ASD-related SRS dataset to date in order to identify any potential redundancy of features in this measure and to explore the linearity of the ASD diagnostic problem space. This work has implications for the replaceability of features when privacy-preserving mechanisms are applied in digital diagnostics and suggests that ASD is a slightly nonlinear phenotype with respect to the behaviors measured by the SRS.

## 2. Methods

### 2.1. Data Sources

Data were aggregated from 7 sources: Autism Genetic Resource Exchange (AGRE),^11^ Autism Consortium (AC), National Database for Autism Research (NDAR),^12^ Simons Simplex Collection (SSC),^13^ Simons Variation in Individuals Project (SVIP),^14^ Autism Speaks (MSSNG), and Autism Genome Project (AGP).^15^ In total, the dataset contains 16,527 individuals with the SRS Child/Adolescent questionnaires completed: 10,004 cases and 6,523 controls. The minority class (controls) was randomly upsampled to achieve class balance. 11,358 individuals are male and 5,169 are female. We note that the risk of ASD has long been noted to affect more males than females, explaining the gender imbalance in the datasets. We did not find any significant difference in demographics or SRS severity between the datasets we used.

### 2.2. Preprocessing

We analyzed data from the Social Responsiveness Scale (SRS), which is a 65-item questionnaire filled out by a caregiver about their child.^4^ The answers to the questions are categorical ordinal variables with a value of 1, 2, 3, or 4. Increasing numbers correspond to behaviors either more or less indicative of social responsiveness, depending on the question.

We used two sets of labels on the same input SRS data for prediction: (1) the diagnosis that would be arrived at using the SRS measure alone (we refer to this as the ‘SRS-derived ASD diagnosis’) and (2) the diagnosis that was provided within the dataset (we refer to this as the ‘dataset-provided diagnosis’). Due to the differences in diagnostic labeling across datasets, we used a list of keywords corresponding to the ‘ASD’ class (e.g., ‘autism’, ‘ASD’, and ‘Asperger’) across the sources as well as another set of keywords corresponding to the ‘not ASD’ class (e.g., ‘control’ and ‘neurotypical’) to arrive at the ‘dataset-provided diagnosis’.

### 2.3. Feature Selection Analysis

In order to test the robustness of the selected features, we applied three different feature selection methods:

(1) *Filter methods:* Univariate filter feature selection methods consider each feature independently and measure the correlation between each feature and the outcome variable. We used the Mutual Information (MI) score. MI is a measure of the dependence between a question (feature) and the clinical ASD classification,^16–18^ quantifying the degree of information gain brought upon by a particular feature.
(2) *Wrapper methods:* Wrapper methods treat the feature selection process as a search problem. We applied a popular wrapper method, recursive feature elimination (RFE), which consists of removing the weakest feature and fitting a model until the desired number of features is achieved. We used a Support Vector Machine (SVM) for the RFE procedure and removed a single feature at each step.
(3) *Embedded methods:* We used the importance scores from a decision tree classifier. The feature importance weights were used to select top features. The same random state was used across all runs of the decision tree.

The reduced feature spaces were used to train a neural network classifying ASD from controls (see section 2.5). All feature selection was performed using the *scikit-learn*^19^ library in Python.

### 2.4. Dimension Reduction

We applied 3 separate dimension reduction techniques: Principal Component Analysis (PCA), t-Distributed Stochastic Neighbor Embedding (t-SNE), and a denoising autoencoder. PCA and t-SNE were implemented using *scikit-learn*. No t-SNE hyperparemeter tuning was performed; the default *scikit-learn* hyperparemters were used for t-SNE (1000 iterations, a perplexity of 30, and a learning rate of 200). To create the denoising autoencoder, we used a dense fully-connected architecture. The ‘encoder’ half of the neural network contained 65 input nodes (corresponding to each SRS question), followed by hidden layers of size 32, 16, 8, and *N*, respectively. Here, *N* represents the number of dimensions we aimed to reduce to. The ‘decoder’ half of the neural network mirrored the ‘encoder’ half, with hidden layers of size 8, 16, 32, and 65 following the encoded layer, respectively. The denoising autoencoder was implemented in TensorFlow^20^ via the Keras^21^ Python library. The low-dimensional representations were used to train a neural network classifying ASD from controls (see section 2.5).

### 2.5. Multi-Layer Perceptron (MLP) Classifier

To determine the minimum number of questions that are needed to predict ASD class from SRS-derived information, we used the top-ranking questions for all three feature selection methods as inputs into a dense neural network. We compared performance using the top *N* questions, with values of *N* ∈ {1, 2, 3, 4, 5, 6}. To determine the number of dimensions needed to represent the data, we also evaluated classifier AUC scores with *M*-dimensional representations of the data using PCA, t-SNE, and the denoising autoencoder, with values of *M* ∈ {1, 2, 3}. In all cases, the deep learning classifier was trained and evaluated using 10-fold cross validation.

The MLP neural network used for evaluation was implemented in Keras^21^ using a TensorFlow^20^ backend. The network consisted of 1 or more fully-connected hidden layers with dropout applied to each hidden layer. In addition to parameterization of the number of hidden layers, hyperparameter optimization was conducted via Bayesian optimization using Hyperopt.^22^ Hyperparameter selection included uniform values between 0.0 and 1.0 for dropout rate, fully-connected layers with possible sizes *∈* {8, 16, 32, 64, 128, 256, 512, 1024}, L2 regulation rate at each hidden layer ∈ {0, 0.001, 0.005, 0.01, 0.05, 0.1}, number of epochs trained ranging from 1 to 10, and using one of { RMSProp, stochastic gradient descent, Adam^23^ optimization } for training of model weights. Binary cross-entropy was used for the loss function.

## 3. Results

### 3.1. Feature Selection Analysis

The features with the highest importance scores using filter, wrapper, and embedded feature selection to predict SRS-derived ASD diagnoses are listed in Table 1. Similarly, the highest-rated questions for predicting dataset-provided diagnoses across all feature selection methods are in Table 2. While feature importance rankings are heavily dependent on the metric used, question 37 (relating to peers) consistently has the highest feature importance out of all SRS questions for predicting SRS-derived ASD diagnosis while question 35 (trouble keeping up with conversational flow) consistently appears in the top-2 features for predicting dataset-specific diagnoses.

Table 3 illustrates the accuracy of the MLP classifier for predicting SRS-derived ASD diagnosis when adding features from the top-ranked list of questions using all 3 feature importance metrics. Table 4 contains the same information for predicting dataset-provided ASD diagnosis. Using only the single top-rated question results in an AUC of over 92% when predicting the SRS-derived diagnosis and an AUC of over 83% when predicting the dataset-provided diagnosis. Using the top-three questions results in an AUC of 97% or higher when predicting the SRS-derived diagnosis and an AUC of 86% or higher when predicting the dataset-provided diagnosis. When using all questions, the AUC is 99.7% for the SRS-derived diagnosis and 90.0% for the dataset-provided diagnosis.

### 3.2. Dimension Reduction

**Fig. 1.**
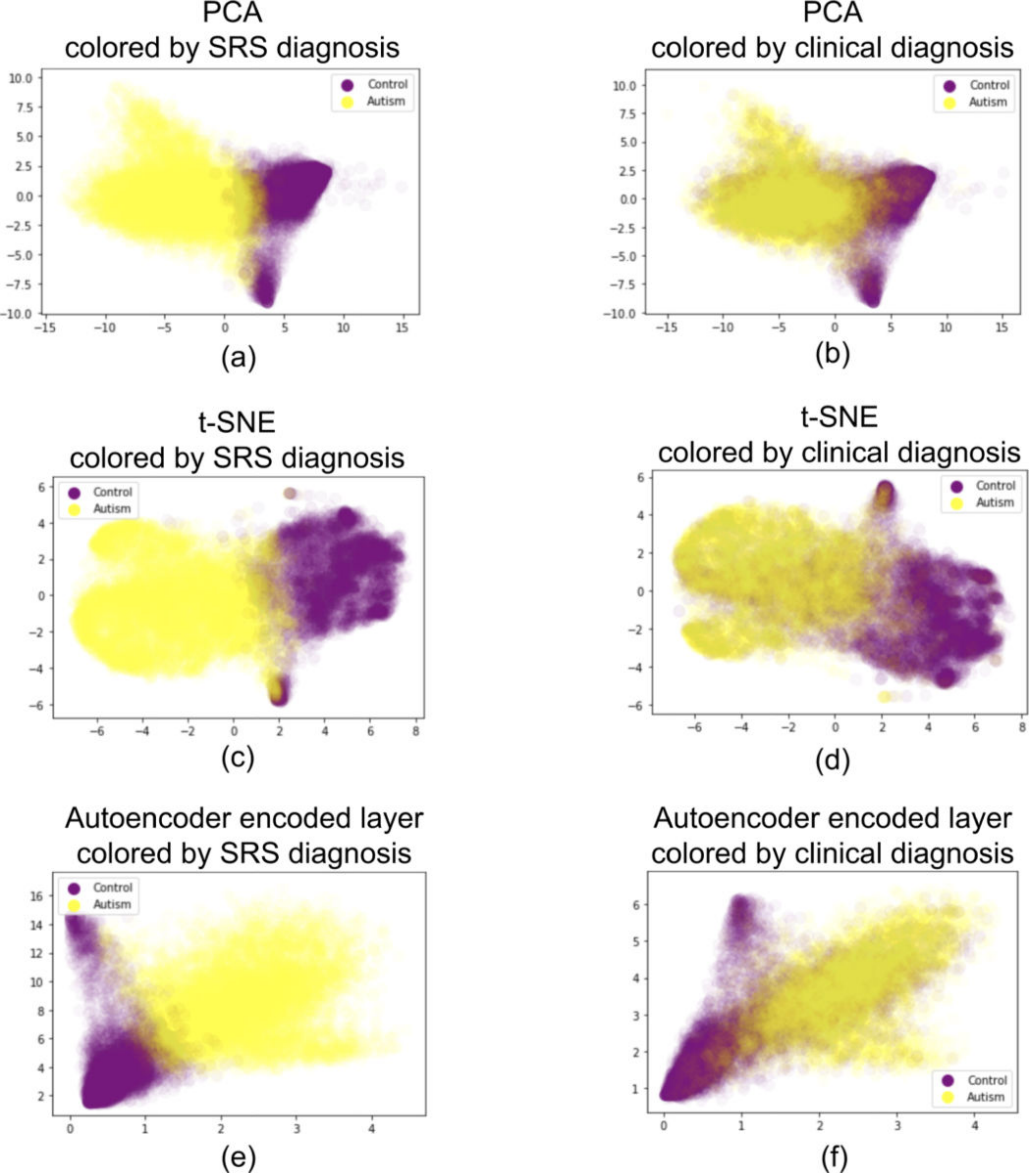
(a and b) Principal Component Analysis (PCA), (c and d) t-Distributed Stochastic Neighbor Embedding (t-SNE), and (e and f) a 2-dimensional encoding using a denoising autoencoder with a middle layer of size 2 on the answers to the 65 questions of the Social Responsiveness Scale (SRS). (b, d, and f) There remains a clear but more noisy separation between cases and controls when coloring by dataset-provided diagnosis.

Figure 1 shows the separation of control (purple) from ASD (yellow) in 2 dimensions for PCA, t-SNE, and the denoising autoencoder, colored by both SRS-derived diagnosis (a, c, and e) and dataset-specific diagnosis (b, d, and f). Even when reducing the SRS space to only 2 dimensions, there is a clear separation between the 2 classes across all techniques.

In order to determine the lowest number of dimensions that the questions can be reduced to while still maintaining high diagnostic accuracy, we evaluated classifier AUC across different numbers of dimensions. Tables 5 (for SRS-derived diagnoses) and 6 (for dataset-provided diagnoses) show the AUC, precision, and recall when predicting ASD diagnosis when reducing to 1, 2, and 3 dimensions using PCA, t-SNE, and the denoising autoencoder.

The baseline AUC of the classifier using all 65 questions of the SRS as features is 99.8% when predicting the SRS-derived ASD diagnosis and 90.3% when predicting the dataset-provided diagnosis. When reducing the dimension to only 1 feature using all three dimension reduction techniques, the AUC is still above 99% when predicting the SRS-derived diagnosis and above 84% when predicting the dataset-provided diagnosis. Increasing the number of dimensions beyond 1 improves the AUC only marginally. Notably, the denoising autoencoder outperforms PCA and t-SNE for the dataset-provided diagnosis when using a low number of dimensions.

### 3.3. Feature Redundancy and Correlation

We analyze redundancy of features by calculating the Spearman correlation between each of the 65 SRS questions. The mean correlation of the 66 possible pairwise-correlations between all distinct questions in the set of questions that appear in the top-6 rated features for at least one of the feature selection methods (questions 8, 12, 13, 16, 24, 29, 33, 35, 37, 44, 58, and 59) is 0.506 (SD = 0.201). By contrast, the mean correlation of the set of all non-identical pairwise correlations is 0.368 (SD = 0.126). A two-sided Welch’s t-test performed between these two sets of correlations is statistically significant (*t* = 5.566, *p* < 0.001). This difference in mean correlation appears in all 7 datasets we aggregated (AGP: *t* = 4.058, *p* = 0.001; AGRE: *t* = 4.865*, *p* <* 0.001; AC: *t* = 4.819, *p* < 0.001; MSSNG: *t* = 5.153, *p* < 0.001; NDAR: *t* = 6.106, *p* < 0.001; SVIP: *t* = 6.000, *p* < 0.001; SSC: *t* = 5.564, *p* < 0.001).

## 4. Discussion

The selected features from the SRS indicate new areas of exploration for ASD diagnostics. Duda et al. identified a similar yet slightly different set of SRS questions7 for distinguishing ASD from ADHD using the mutual information metric (ranked from most to least important): trouble with the flow of normal conversation (question 35), difficulty with changes in routine (question 24), appropriate play with peers (question 22), difficulty relating to peers (question 37), atypical or inconsistent eye contact (question 16), and being regarded as ‘odd’ by other children (question 29). It is interesting to note that the top-3 ranking features for distinguishing an SRS-derived ASD diagnosis from a control using mutual information (Tables 1 and 2)-namely, trouble with the flow of normal conversation, difficulty relating to peers, being regarded by other children as ‘odd’-appear in the list of top-ranking features for distinguishing ASD from ADHD as reported by Duda et al. This provides validation of Duda et al.’s work using a larger dataset. This also suggests that these features may distinguish neurotypical children from children with ASD, although further work is needed. In addition, the understanding of cause and effect appears as a new high-ranking feature in this study, hinting at the possibility that the understanding of how events relate to each other might be critical to social behavior.

Due to the general nature of the SRS questions, it is likely that the top questions are not specific to ASD-that is, high sensitivity but low specificity. Due to the feature overlap with Duda et al.^7^ when distinguishing ASD from ADHD, many of the features are able to successfully identify behaviors specific to ASD and one of its common co-occurring disorders, ADHD. Future work is required to determine the predictive power of these questions for other behavioral disorders. The procedures described, while here applied towards ASD, could be generalized and applied to other mental health conditions where electronic medical record (EMR) information is stored.

Such a large dataset has not been used except by Paskov^24^ when exploring low dimensional representations of SRS and other ASD questionnaires for imputation. The increased size of the data permitted an exploration of dense neural networks with ReLU activation, a method traditionally suited for large data.

The low ranking of features in the decision tree that appear as high ranking ASD features for MI and RFE suggest that there is high feature redundancy among SRS questions. In particular, trouble keeping up with conversational flow and trouble relating to peers seem to have high feature redundancy, but these behaviors are distinct from being regarded as ‘odd’. This hints that certain features that are obfuscated by privacy-preserving mechanisms could be replaced by the extraction of other non-obfuscated features. Instead of digitally tracking trouble relating to peers, which might require consent or assent from any peer that the child in question interacts with when using a digital diagnostic or intervention, the device could instead track trouble keeping up with conversational flow, which only requires consent from the child in question. Future work is required to explore additional redundancy of features according to the SRS in addition to redundancies present in other measures of ASD severity.

The dimension reduction analysis provides initial insight into the linearity of the ASD phenotype, as the performance of the classifier across the three methods yielded similar performance, indicating that the ASD phenotype can be successfully distinguished with linear methods. However, the superior performance of the autoencoder suggests that the phenotype is at least slightly nonadditive. We also point out the limitations of using t-SNE: in particular, the t-SNE representation was not fit to the testing set, and the t-SNE hyperparameters were arbitrarily chosen. As the dataset-provided diagnoses are much more noisy, this work provides support for the potential of denoising autoencoders to produce low-dimensional representations of noisy high-dimensional data. As denoising autoencoders do not require hyperparemter tuning (unlike t-SNE), the method may also be more convenient and computationally efficient than t-SNE in some cases, including the use case presented in this paper.

## 5. Future Outlook

Precision medicine therapies for ASD are beginning to track longitudinal behavioral phenotype changes for measuring treatment outcomes. The feature reduction conducted here sets the stage for future work exploring mechanisms of replacement of the behavioral measurements collected in digital monitoring tools based on redundancy of information. Beyond single time-point diagnostics, the results of the present study can inform areas of focus for future digital phenotyping^25^ efforts for ASD. At-home digital therapies for ASD^5,6,26^ could benefit from targeted tracking of behavioral features in order to provide customized digital therapies to the child. Dual-purpose digital therapies aimed at simultaneous data capture and intervention^27–30^ could focus the area of target towards capturing salient behaviors. Furthermore, crowdsourcing has been shown to be an effective technique for acquiring near-clinical grade answers to instrument-derived diagnostic questions.^31–33^ When crowdsourcing the acquisition of answers to questions for video-based ASD diagnostics in this way, replacing questions that have related diagnostic power can enable shorter and customizeable feature sets.

Because a single dimension was sufficient for compressing ASD data, it is feasible to imagine the development of more efficient scoring schemes with data-driven methods. The ‘map’ of ASD appears to be at least slightly nonadditive, suggesting more work with nonlinear models for classification. Furthermore, current rule-based scoring schemes for SRS and other ASD instrument data could be replaced by supervised (via feature selection) or unsupervised (via dimension reduction) approaches.

## Figures and Tables

**Table 1.** The SRS questions with the highest feature importances for predicting the **SRS-derived ASD diagnosis**. Because Recursive Feature Elemination (RFE) does not weight the selected features, we display the values of *N* for which the question appears in the top-*N* for values of *N* up to 6.

SRS Question	Mutual Information (MI) Score (Rank)	RFE Features to Select	Decision Tree (Rank)

Relating to peers (37)	0.383 (1)	1, 4, 5, 6	0.604 (1)
Trouble keeping up with conversation flow (Q35)	0.355 (2)	N/A	0.005 (13)
Regarded by other children as odd (Q29)	0.339 (3)	2, 6	0.002 (47)
Socially awkward, even when trying to be polite (Q33)	0.333 (4)	3	0.006 (11)
Bizarre mannerisms (Q8)	0.332 (5)	3, 4, 5, 6	0.030314 (4)
Trouble understanding cause and effect (Q44)	0.324 (6)	2, 4, 5, 6	0.099 (2)
Difficulty with changes in routine (Q24)	0.292 (9)	3, 4, 5, 6	0.021 (5)
Communication of feelings to others (Q12)	0.134 (47)	5	0.005 (14)
Focuses on details rather than the big picture (Q58)	0.216 (23)	6	0.004 (22)
Either avoids or has unusual eye contact (Q16)	0.287 (20)	N/A	0.035 (3)

**Table 2.** The SRS questions with the highest feature importances across selection methods for predicting the **dataset-specific ASD diagnosis**. Because Recursive Feature Elemination (RFE) does not weight the selected features, we display the values of *N* for which the question appears in the top-*N* for values of *N* up to 6.

SRS Question	Mutual Information (MI) Score (Rank)	RFE Features to Select	Decision Tree (Rank)

Trouble keeping up with conversation flow (Q35)	0.224 (1)	1, 2, 3, 4, 5, 6	0.391 (1)
Relating to peers (Q37)	0.205 (2)	6	0.007 (51)
Regarded by other children as odd (Q29)	0.204 (3)	2, 3, 4, 5, 6	0.057 (2)
Trouble understanding cause and effect (Q44)	0.203 (4)	4, 5, 6	0.010 (16)
Trouble with conversational turn taking (Q13)	0.179 (5)	N/A	0.010 (20)
Either avoids or has unusual eye contact (Q16)	0.172 (8)	3, 4, 5, 6	0.024 (3)
Bizarre mannerisms (Q8)	0.178 (6)	N/A	0.006 (56)
Is overly suspicious (Q59)	0.002 (65)	5, 6	0.005 (63)
Repetitive behaviors (Q50)	0.126 (19)	N/A	0.019 (4)
Repetitive behaviors (Q57)	0.045 (56)	N/A	0.012 (5)

**Table 3.** The AUC, precision (prec.), and recall (rec.) of a dense neural network predicting the **SRS-derived ASD diagnosis** trained on the top-ranking features of the 65-item SRS questionnaire using each feature selection technique.

Number of Questions (Features)	Mutual Information AUC / Prec. / Rec.	RFE AUC / Prec. / Rec.	Decision Tree AUC / Prec. / Rec.

1	0.928 / 0.900 / 0.928	0.928 / 0.900 / 0.928	0.928 / 0.900 / 0.928
2	0.961 / 0.947 / 0.906	0.955 / 0.912 / 0.919	0.962 / 0.943 / 0.906
3	0.971 / 0.919 / 0.953	0.975 / 0.932 / 0.938	0.973 / 0.939 / 0.933
4	0.974 / 0.937 / 0.938	0.979 / 0.941 / 0.944	0.980 / 0.944 / 0.943
5	0.980 / 0.941 / 0.941	0.982 / 0.939 / 0.948	0.984 / 0.950 / 0.951
6	0.983 / 0.944 / 0.949	0.985 / 0.949 / 0.951	0.987 / 0.950 / 0.961

**Unaltered**		0.997 / 0.972 / 0.979	

**Table 4.** The AUC, precision (prec.), and recall (rec.) of a dense neural network predicting the **dataset-provided ASD diagnosis** trained on the top-ranking features of the 65-item SRS questionnaire using each feature selection technique.

Number of Questions (Features)	Mutual Information AUC / Prec. / Rec.	RFE AUC / Prec. / Rec.	Decision Tree AUC / Prec. / Rec.

1	0.836 / 0.750 / 0.774	0.836 / 0.727 / 0.843	0.836 / 0.724 / 0.836
2	0.866 / 0.730 / 0.882	0.870 / 0.734 / 0.907	0.870 / 0.735 / 0.899
3	0.874 / 0.735 / 0.902	0.876 / 0.738 / 0.905	0.876 / 0.736 / 0.909
4	0.879 / 0.739 / 0.912	0.881 / 0.740 / 0.917	0.880 / 0.741 / 0.907
5	0.880 / 0.739 / 0.916	0.880 / 0.740 / 0.911	0.882 / 0.737 / 0.920
6	0.881 / 0.736 / 0.924	0.884 / 0.742 / 0.923	0.886 / 0.745 / 0.914

**Unaltered**		0.900 / 0.754 / 0.921	

**Table 5.** The AUC, precision (prec.), and recall (rec.) of a dense neural network predicting the **SRS-derived ASD diagnosis** and trained on lower dimensional representations of the 65-item SRS questionnaire via PCA, t-SNE, and the middle encoded layer of a denoising autoencoder.

Dimension	PCA AUC / Prec. / Rec.	t-SNE AUC / Prec. / Rec.	Autoencoder AUC / Prec. / Rec.

1	0.9975 / 0.9778 / 0.9719	0.9871 / 0.9702 / 0.9356	0.9975 / 0.9828 / 0.9740
2	0.9975 / 0.9769 / 0.9759	0.9934 / 0.9766 / 0.9514	0.9974 / 0.9503 / 0.9950
3	0.9979 / 0.9739 / 0.9739	0.9920 / 0.9734 / 0.9415	0.9975 / 0.9818 / 0.9730

**Unaltered**		0.9979 / 0.9799 / 0.9884	

**Table 6.** The AUC, precision (prec.), and recall (rec.) of a dense neural network predicting the **dataset-provided ASD diagnosis** and trained on lower dimensional representations of the 65-item SRS questionnaire via PCA, t-SNE, and the middle encoded layer of a denoising autoencoder.

Dimension	PCA AUC / Prec. / Rec.	t-SNE AUC / Prec. / Rec.	Autoencoder AUC / Prec. / Rec.

1	0.8717 / 0.7272 / 0.9097	0.8448 / 0.7246 / 0.9356	0.9017 / 0.7304 / 0.9373
2	0.8727 / 0.7206 / 0.9110	0.8821 / 0.7650 / 0.9157	0.9016 / 0.7193 / 0.9564
3	0.8813 / 0.7306 / 0.9150	0.8788 / 0.7542 / 0.8934	0.9021 / 0.7304 / 0.9373

**Unaltered**		0.9034 / 0.7673 / 0.9161	
